# The Ethical Basis of Severity as a Priority Setting Criterion in Healthcare—Egalitarian or Prioritarian?

**DOI:** 10.1007/s11673-025-10472-1

**Published:** 2025-09-11

**Authors:** Niklas Juth, Erik Gustavsson, Lars Sandman

**Affiliations:** 1 Centre for Research and Bioethics, Department of Public Health and Caring Sciences, Uppsala University, Husargatan 3, 752 37 Uppsala, Sweden; 2 Division of Philosophy and Applied Ethics, Department of Culture and Society, Linköping University, Campus Valla, Key, Ingång Key, 581 83 Linköping, Sweden; 3 National Centre for Priorities in Health, Department of Health, Medicine and Caring Sciences, Linköping University, Hus 511, Ingång 76 Eller 78, Plan 13, Campus US, Lasarettsbacken, 581 83 Linköping, Sweden

**Keywords:** Egalitarianism, Healthcare, Prioritarianism, Priority setting, Severity

## Abstract

This article discusses the most plausible moral basis for using severity as a priority setting criterion in healthcare: prioritarianism or egalitarianism. We argue that prioritarianism is superior, since egalitarianism has several problems that prioritarianism avoids. We have elaborated three such problems. First, egalitarianism arguably needs a non-equality-based reference level in order to determine the magnitude of severity. Second, it has the problem of irrelevant alternatives: the assessment of the severity of one person’s illness varies depending on the condition of other persons, even when their health status has not changed. Third, egalitarianism introduces excessive complexity, as it must explain what aspects of inequality matter, and why, in relation to illness severity. By contrast, prioritarianism has some benefits that egalitarianism lacks: it aligns theoretically with the concept of severity as a priority setting criterion in healthcare, and it explains why we always have a pro tanto reason to improve someone’s health without having to rely on other theories. In the end, if equality of health matters, it is arguably not because of its connection to severity.

## Introduction

Priority setting is unavoidable in any healthcare system as it encompasses all decisions on the allocation of healthcare resources, including which order patients are tended to. In publicly funded healthcare systems, for instance some European ones, there are officially formulated criteria for priority-setting decisions. A common criterion is cost-effectiveness, that is, healthcare resources should be allocated in such a way that we get as much health or health-related quality of life from the system as possible (we shall use health as short for health-related quality of life throughout the manuscript). However, considerations of cost-effectiveness are often tempered by considerations of just distribution.

In some countries, for instance, Sweden, Norway, the Netherlands, and the United Kingdom, these justice-based considerations are primarily formulated in terms of severity of illness (henceforth severity for simplicity) (Sandman and Juth 2024). The general idea is that the more severe an illness is, the higher the priority assigned to the patient group or patient with that illness.^1^ In practice, severity then functions as a modifier for what is considered an acceptable cost-effectiveness threshold for treatments: the more severe a condition is deemed to be, the larger the cost per health gain society is willing to pay. It is severity in this sense that we focus on: as a justice-based modifier for priority setting within healthcare.

Although severity is an important criterion for priority setting, it remains unclear and unexplored how the criterion should be understood more precisely (Barra et al. 2020). One explanation for this is that there is no generally accepted moral basis for severity as a criterion for priority setting in healthcare (Barra et al. 2020). Hence, there is no generally accepted idea of *why* we should care about severity in the first place when it comes to just allocation of healthcare resources. So, what kind of moral basis for severity as a priority setting criterion in healthcare is most plausible? In this article, we explore two possibilities, egalitarianism and prioritarianism, and argue that the most plausible principle is prioritarian rather than egalitarian.^2^ This exploration is worthwhile in its own right and contributes to the ongoing ethical debate among scholars as to whether prioritarian (Barra et al. 2020) or egalitarian considerations (Kamm 2002; Nord and Johansen 2014) are the proper basis for severity as a priority setting criterion.

The scope of the argument should be made clear at the outset: although we argue that prioritarianism is the more plausible basis for severity as a priority setting criterion in healthcare, we do not say that egalitarian considerations should be altogether discarded in healthcare allocation. The claim in this paper is merely that severity as a healthcare priority setting criterion does not have an egalitarian basis but, rather, a prioritarian one. This is compatible with the position that egalitarian considerations should make a difference for healthcare allocations independently of severity. This also means that the criticism levelled against egalitarianism in this manuscript is not against egalitarianism in general but specifically against egalitarianism as a basis of assessing severity, nothing more. Therefore, the arguments against egalitarianism presented are not generic arguments against egalitarianism but specific and novel ones against egalitarianism as applied in this context.

We will begin by specifying the notion of severity of illness as a priority setting criterion. Then we will explain the relationship between severity, on the one hand, and prioritarianism and egalitarianism respectively, on the other. We then address the substance of our argument, presenting three problems with egalitarianism that prioritarianism avoids: the problem of needing a non-relational reference level, the problem of irrelevant alternatives, and the problem of excessive complexity. We then present the benefits of grounding severity in prioritarianism rather than egalitarianism. We conclude that prioritarianism more plausibly constitutes a moral basis for using severity of illness as a priority setting criterion.

## Severity

We make the following assumptions regarding the concept of severity as a priority setting criterion (see also Jølstad and Juth 2022):

- Severity is a function of how bad the illness is for the person, i.e., the negative effect the illness has for the person. By extension, the worse off the illness makes the person, the more severe the illness is. And, conversely, the more severe an illness is, the worse off the person is (in terms of illness). This means that severity in this context only deals with health or health-related quality of life (since we are dealing with healthcare allocation only), not quality of life in general (see below). This is based on the premise that healthcare as a practice is concerned with a more narrow conception of what is good for people, rather than broader theories about what makes a person’s life go well.
- Severity is in principle a graded concept: people can have greater or lesser severity. In making our arguments, we assume that various degrees of severity can be meaningfully represented on some (at least ordinal) scale (although, in practice, such scale may have problems of assessing and comparing the different components involved in such a state). Throughout the article, we presuppose such a graduated conception of severity (although, when there are no health issues whatsoever, it makes sense to talk about no severity at all, see below).

It is also important to note what kind of currency or object of allocation we are examining in this context. When we discuss healthcare allocations, we will, for simplicity, use the term *health* as the name of our currency. With health we imply something in terms of health-related quality of life and length of life, e.g., captured by different measures like quality adjusted life years (QALYs). We remain neutral on exactly what measurement or conception of health is presupposed, but we do assume that prioritarianism and egalitarianism (in this context) agree that both length of life and (health-related) quality of life matter.

## Severity According to Prioritarianism and Egalitarianism

*Prioritarianism* holds that “[b]enefiting people matters more the worse off those people are” (Parfit 2000, 19). The idea is that the moral importance of health gain is greater the worse a person’s health status is. This view assumes that health (or, again, whatever you take to be the relevant currency) has diminishing marginal *moral* importance: the better your health already is, the less moral weight further improvements carry. In this context, we are using prioritarianism in the sense of a strict concave relationship between health and moral importance (Adler 2022). The precise shape of this function—that is, how quickly moral importance diminishes as health increases— depends on how much weight we put on improvements on the various levels. We will not take a stand on that issue here, since it is not important for the argument we want to pursue (for more on this, see Herlitz and Horan 2016).

Moreover, and in this context especially important to note, the idea that improvements in health are of diminishing moral importance the better off someone is, and conversely of increasing importance the worse off someone is, should be understood in a *non-relational* way: it is the absolute level of health that matters for the value of health improvements at that level, regardless of how well or badly off other patients are.

What would prioritarianism as a moral basis for severity in this context imply? It would imply that being of ill health is worse (has a higher severity) than being of moderate health. It would mean that moving someone from ill health to moderate health has a higher moral value than moving someone from moderate health to good health, even if we assume that the improvements of health are of equal magnitudes in both cases. So, in healthcare, according to a prioritarian account of severity, we should be prepared to “waste” health (from a purely health maximizing point of view) in order to tend more to those worse off. (An illustration of prioritarian kinds of distributional theory that uses health-related social welfare functions is developed in Norheim et al. 2021.)

*Egalitarianism*, as understood in this paper, is the view that “[i]t is in itself bad if some people are worse off than others” (Parfit 2000, 84). We are, then, only referring to what is often called *telic egalitarianism* when we use the term egalitarianism (Hirose 2015).^3^ Telic egalitarianism is also a pluralist view in claiming that what matters is not only equality but also the total or average sum of value: the more the better (see below). In contrast to prioritarianism, egalitarianism is a *relational* view of final value: the value of a state of affairs depends, partly, on how well off or badly off individuals are in relation to each other. So, in this case, at least one reference level for severity is the situation when there are no health differences between the persons in the reference population (more on various such possibilities below).

There are problems that apply equally to both egalitarian and prioritarian accounts of severity and for that reason can be set aside as not lending support to either side of the debate. First, prioritarianism needs to specify how much more improvements of those worse off should weigh in comparison to those better off. But this is analogous to the problem for egalitarianism regarding how much equality should weigh in relation to health improvements (or whatever currency one is dealing with). Another issue is whether prioritarianism should be applied to whole lives, segments of lives, or momentarily (Ottersen 2013). But since the same problem accrues to egalitarianism (Temkin 1993), we will ignore this in the following analysis (see Sandman and Juth 2024 for a recent analysis of the temporality of severity).

As said in the outset: we will now present three problems or challenges for severity as a priority setting criterion in healthcare based on egalitarianism and, hence, understood in the relational way, problems that severity based on prioritarianism would deal better with.

## Severity Based on Egalitarianism: The Problem of Needing a Non-Relational Reference Level

In this section, we focus on the challenge for egalitarians of identifying a reference level that is consistent with an egalitarian spirit. For prioritarians, this is less of a challenge, since they can reasonably assume that the reference point for assessing absolute levels is a threshold above which people have no, or no significant, health problems. Of course, egalitarians could make the same assumption as a basis for severity, but doing so would come at the cost of conceding partial ground to non-relational conceptions of justice, such as prioritarianism. Let us elaborate.

We have already mentioned one possibility for egalitarians: a reference population. To spell this out, let us start with a simple and a strawman version of egalitarianism, according to which equality is the *only* thing that matters. On this view, it is bad in itself that some people are worse off than others. Egalitarianism therefore ascribes final value to equality: it holds that equality is valuable for its own sake rather than for any good consequences that might flow from it. Applied to severity, this would mean that severity is defined solely in terms of how a person’s health compares with that of a reference population. When there are no health differences in that population, there is no severity.

This leads to counter-intuitive results. Assume a group of people where everyone is equally healthy. The severity, from the egalitarian viewpoint, will be the same—none. But obviously, there is huge difference if the individuals in the group are in terrible health or very good health—being in terrible health is much worse than being in very good health. Failing to account for that is devastating for a rationale for severity. Accordingly, it seems clear that severity must have *some* other reference —a baseline indicating zero severity—so that the genuinely negative state of being in poor health can be distinguished from the positive state of being in good health. It is reasonable to assume that a person may have no, or no significant, illnesses whatsoever—in which case it is not meaningful to talk about severity (unless in the sense of “no severity at all”). So, whether the rationale behind severity of illness is egalitarian or prioritarian, at some point there are no claims whatsoever to be prioritized on grounds of severity. However, to conceptualize the reference level in terms of other people is a non-starter. This approach ignores a basic idea behind severity: the need to distinguish differences in the impact of illness. Hence, as stated above, we turn instead to a more plausible egalitarian theory, i.e., some version of so-called telic egalitarianism.

Telic egalitarians are value pluralists: they are claiming that both *improving* and *equalizing* people’s well-being have final value and that some improvements are worth foregoing in order to achieve greater equality. However, they recognize limits to how much equality should be bought at this cost (Hirose 2015, 64–67).^4^ Analogously, an egalitarian account of severity as a priority setting criterion in healthcare could hold that severity is partly determined by how badly off one is in relation to a reference threshold, thereby avoiding the absurdity highlighted above. However, this account would still maintain that severity is also partly determined by comparison to others, maintaining the egalitarian core. On this view, the telic egalitarian would claim that it is worse for all individuals in a population to be in terrible, rather than good, health—even if the “equality-component” of severity is zero in both cases.

However, this egalitarian account succeeds only to the extent that it cedes ground to prioritarianism. It acknowledges that prioritarianism is correct in holding that a non-relational reference level partly determines severity, and not only relationship to others. We will refer to this challenge as *the problem of needing a non-relational reference level.*

## Severity Based on Egalitarianism: The Problem of Irrelevant Alternatives

There is a much more serious problem for egalitarian or relational accounts of severity: the severity of your condition will vary according to the health level of others, even if *you* remain on the same level of health. Moreover, those whose health remains the same in two patterns of distribution with regard to how badly or well off they are could nonetheless have more or less severe illness depending on how others fare. This is part and parcel of the pluralistic egalitarian notion, since final value of equality is relationally determined: it hinges on how well or badly off you are in comparison to others.^5^

Let us illustrate. Assume that someone, Derek, is in ill health (with some severity) while the rest of a population is in very good health (no severity). If the rest of the population drops to the same level of health as Derek, Derek’s situation appears less severe, because his health is now equal, although nothing whatsoever has happened to Derek’s health. If the others get better and bounce back to very good health while Derek’s level of health remains the same, his situation appears more severe, again solely because it is less equal, despite no change in his own health.

These examples illustrate that egalitarianism regarding severity violates the independence of irrelevant alternatives condition (inspired by but not completely identical to Kenneth Arrow’s idea): how severe the illness of one person *P* is should not be affected merely by how others (unrelated to *P*) fare if *P* is not affected at all. It seems to border on a platitude regarding severity: how severe *your* illness is cannot in itself be affected merely by *my* illness getting worse or better. Of course, if you are aware of me getting worse off, you, as a result of this, may get worse off (e.g., get depressed) or better off (e.g., in getting some relief in your suffering to know others are also suffering). But then you *are* affected, and the point does not apply. The point is that, according to egalitarianism, the severity of your illness is (partly) affected by how others (lacking a relevant relation to you) fare, even though your illness has not changed whatsoever, and they are not causing your illness to change in any way.

It may even be the case that, all things considered, someone’s severity may be worsened although their health status improves, for instance, if the health status of others are improved more. For instance, if *P* moves from terrible health to very bad health while the others move from moderate health to very good health then inequality has increased substantially and, according to some ways of measuring the value of inequality, *P* is worse off and, hence, more severely ill, although *P* is, in fact, better off health-wise. This would apply, for instance, if we measure inequality by adding all the differences between all individuals in a population. Once again, this is counter-intuitive to the concept of severity. Prioritarian accounts of severity have no such problems as they determine the level of severity in a non-relational way from the outset.

## Severity Based on Egalitarianism: The Problem(s) of Excessive Complexity

Egalitarians, like prioritarians, must explain *why* severity matters from a moral point of view. On this front, however, egalitarians face more difficulties. One reason is that there are many more versions of egalitarianism than of prioritarianism (see below), and it is not yet clear which version egalitarians generally favour, or which applies specifically to severity.

In his seminal work *Inequality* (1993), Larry Temkin distinguishes twelve different aspects in which an outcome may be better or worse in terms of equality, each with some intuitive appeal. First, we must decide how to measure the magnitude of inequality for those who are worse off. Here, there are three ideas: one can compare them to the average level (AVE), the best off person (BOP), or to all those better off (ATBO). Second, we must decide which groups to include when making this comparison. Again, three options arise: we include only those worst off (maximin principle: MP); we include everyone worse off according to AVE, BOP, or ATBO and add their levels in relation to the chosen measure (additive principle of equality: AP); or we adopt AP but weight the worse-off more heavily (weighted additive principle: WAP).^6^

These two sets of choices can be combined in different ways, producing nine possible approaches. For instance, combining AVE and MP implies that, as more people are brought down to the lowest welfare level (while that level itself remains unchanged), the better the situation becomes in terms of equality, since the average will continuously decrease. In contrast, combining BOP and AP implies that the more people we raise to the top level of the best-off person, ceteris paribus, the better the situation in terms of equality. Hence, deciding which aspects of inequality matter is far from trivial: it directly affects how outcomes are ranked from an egalitarian perspective.

To add to the complexity, one may value pluralism within the egalitarian framework, holding that several of these aspects matter and must be weighed against each other when in conflict. This, indeed, is the approach that Temkin himself favours (Temkin 1993, 77).

This gives rise to three related problems for egalitarianism. First, the egalitarian need to tell us what aspects of equality among those presented are relevant to determine degree of severity of illness and, furthermore, why these are relevant (and not the others). This remains to be done. In the light of the many versions of egalitarianism the challenge seems close to insurmountable.

Second, an alleged way out of this problem would be to adopt the suggested position that all these aspects matter and must be weighed together. However, this solution has serious shortcomings of its own. To start with, the egalitarian still owes us a justification as to why *all* these aspects matter. And not only a justification that makes it clear why we should care about these aspects in healthcare priority setting (the normative challenge) but also one that is tied to the concept of severity of illness (the conceptual challenge).

Moreover, pluralism comes with the cost of indeterminacy (Herlitz 2017). If all these aspects matter and pull in opposite directions in many cases (as we saw above), it may be unclear what we should say about the (dis)value of (in)equality except in the simplest cases—for example, when we move from perfect equality to inequality or when someone is made worse off while no one is made better off. But the first kind of case is in practice extremely unlikely to ever happen, and the second one prioritarianism handles as well with the same result as egalitarianism. As soon as the situation becomes more complicated, for example, when we compare outcomes with different numbers of people, or when the situation of some is made worse, while others improve, it becomes unclear how to compare the different aspects of inequality with each other. Hence, egalitarianism leaves us in the lurch when we need it the most for purposes of healthcare priority setting.

Of course, an egalitarian may try to bypass this difficulty by proposing one of its simplest versions, like BOP combined with MP. This version of egalitarianism would only require us to keep track of the best- and worst-off persons or groups. But the simplicity of this version is bought at the cost of low plausibility, especially if one is motivated by egalitarian considerations to start with: again, why should one only care about the relative positions of the best and worst off and ignore how everyone else fares in relation to each other if one cares about equality? From an egalitarian perspective, it seems that all relationships should matter, so a combination of ATBO and AP would be more appealing and truer to the credo of egalitarianism. Or, perhaps even better, a combination of ATBO and WAP, since that combination would more readily reject gross inequalities. In this case, one has, interestingly enough, added a prioritarian element into egalitarianism, since the differences are weighed. And then we are back at the original problem of difficulty of putting egalitarianism into practice.

## Two Appealing Features of Prioritarianism as a Basis of Severity

Besides the advantages of prioritarianism over egalitarianism already presented, we would like to briefly mention two other features of prioritarianism that make it more attractive.

First, severity and prioritarianism explicitly share the same basic moral intuition: we have stronger reasons to assist people the worse off they are. This is exactly what prioritarianism states. The intuition that this is the case, regardless of how well or badly off *other* people are, seems especially plausible when it comes to severity of illness. As it seems, conceptually, severity of illness is not a relational notion at all: how severely ill someone is should be understood independent from how severely ill others are. Looking at an individual with great suffering about to die shortly due to her illness, we can safely state that she is severely ill just by comparing her condition to a situation in which she does not have any suffering and has a long life ahead—without reference to whether others are better or worse off. Her suffering and life-expectancy (or whatever factors we include in the health currency) will not change merely because other people become more or less healthy. Furthermore, it seems intuitive that, ceteris paribus, the more severe an illness is, the stronger the reason to alleviate it. We conjecture that this conceptual and moral match between severity and prioritarianism explains why the independence-of-irrelevant-alternatives argument elaborated above is forceful.

This also means that prioritarianism, when applied to severity, satisfies a version of the Pigou-Dalton principle: “A pure, non-rank-switching transfer of well-being from someone better off, to someone worse off, leaving everyone else unaffected, is a moral improvement” (Adler and Holtug 2019, 104). A pure transfer here means a transfer of the same amount, which is of course a necessary condition for the Pigou-Dalton principle to be plausible.

Translated into this context, the principle implies that, between two alternative outcomes differing only in that the latter improves the condition of a more severely ill person while worsening that of a less severely ill person (who still remains less ill than the former), the latter outcome should always be preferred in healthcare priority setting—at least if the size of the health change is equal for both parties. In itself, this is appealing basis for just healthcare priorities. Admittedly, this also goes for some—but not all—versions of telic egalitarianism (Adler and Holtug 2019).

Second, prioritarianism implies that any improvement in someone’s health is always a pro tanto reason to prefer that outcome, which is also intuitively appealing. Egalitarianism does not share this advantage, since under some versions an improvement in one person’s condition may increase inequality to such an extent that it counts as a pro tanto reason against the improvement (Adler and Holtug 2019).

## Conclusion

This article has examined which moral framework provides the most plausible moral basis for using severity as a priority setting criterion in healthcare: prioritarianism or egalitarianism. While both have some initial appeal as general theories of justice, we argue that prioritarianism is the stronger foundation for severity. Egalitarianism faces several difficulties that prioritarianism avoids. We have elaborated three in this article. First, egalitarianism also appears to need a non-equality-based reference level in order to determine the magnitude of severity. Second, it is vulnerable to the problem of irrelevant alternatives: the severity of one person’s illness can shift merely because of changes in others’ conditions, even when nothing about that person’s health has changed. Third, there is the problem of excessive complexity, since it must explain which dimensions of inequality matter, and why, in relation to severity of illness.

In contrast, prioritarianism offers advantages that egalitarianism lacks: there is a theoretical match between prioritarianism and severity as a priority setting criterion in healthcare, and prioritarianism explains why we always have a pro tanto reason to improve someone’s health, without appeal to other theories. In the end, if equality of health matters, it is arguably for reason other than severity.

## Data Availability

Not applicable. This study is theoretical/conceptual and does not involve data or material gathered from persons or animals. There is no dataset to be made available. Any questions about the study can be directed to niklas.juth@uu.se.

## Footnotes

1 It is often unclear whether severity is properly attributed to patients or conditions. We will remain neutral on this issue in this context—it has been extensively discussed elsewhere (Gustavsson 2019).

2 There may, of course, be other principles that could serve as the moral basis of severity of illness as a priority setting criterion in healthcare, e.g., sufficientarianism, but that is not the subject of this article.

3 We are therefore excluding, for instance and foremost, Rawlsian egalitarianism and luck egalitarianism. The latter would add to egalitarianism that responsibility for ill health should play a role in priority setting (Björk 2021)—something we ignore in this context. The former, we believe, is untenable as a priority setting criterion in healthcare in the first place, but that is a question for a separate article—see, e.g., Juth 2015. We are also excluding political egalitarianism more broadly understood, e.g., Elizabeth Anderson’s democratic egalitarianism (Anderson 1999).

4 As Hirose notes, a telic egalitarian does not have to be a value pluralist in order to claim that unequal distributions are worse than equal, if one favours the aggregative view (Hirose 2015, 76–80). But as far as we can see, the position regarding severity presented here is still open to a proponent of the aggregative view.

5 The problem of irrelevant alternatives when it comes to severity is of course an implication of egalitarian theories not fulfilling the separability condition (Adler and Holtug 2019, 104; Norheim et al. 2021, 270).

6 Moreover, we have the aspects of Gratuitousness (where the value of inequality is determined by how much it costs to eliminate it), Social inequality (where the value of inequality is determined by it being an effect of social institutions), and Deviation (where the value of inequality is determined by how far the situation is from full equality, e.g. Gini-coefficient) (Temkin 1993, 51).
